# Temporal reward discounting in children, adolescents, and emerging adults during an experiential task

**DOI:** 10.3389/fpsyg.2014.00711

**Published:** 2014-07-08

**Authors:** Anouk Scheres, Chandra Tontsch, Allison L. Thoeny, Motofumi Sumiya

**Affiliations:** ^1^Department of Psychology, University of ArizonaTucson, AZ, USA; ^2^Behavioural Science Institute, Radboud UniversityNijmegen, Netherlands; ^3^Division of Cerebral Integration, National Institute for Physiological SciencesOkazaki, Japan

**Keywords:** adolescence, reward, temporal reward discounting, delay discounting, impulsivity, self-control

## Abstract

The goal of this study was to examine age effects on the ability/willingness to wait for large rewards in a real temporal reward discounting task from childhood to adulthood. Therefore, a real temporal discounting (TD) task was administered to children aged 6–12 (*n* = 39), adolescents aged 13–17 (*n* = 28), and young adults aged 18–19 (*n* = 55). Findings indicated that the cross-sectional development of TD followed a quadratic pattern across age groups, with adolescents choosing more often than children and adults to wait for the large delayed reward, resulting in reward-maximization. Various interpretations of this finding were offered, including a focus on reward maximization despite an immature ability to exert self-control, and flexible self-control which was high during this task as a result of strong motivation to maximize financial gains.

## Introduction

Trading the anticipated benefits and costs of two choice options at different points in time is an important skill that we need to navigate through life: balancing the pursuit of future goals with enjoyment of the present moment, choosing between a high-fat dinner which you may enjoy and a perhaps less-preferred but healthier salad, deciding whether to save money for retirement or spending most of it now. When this balancing of short-term smaller gains and long-term larger gains is measured in laboratory settings, this is typically done by the use of temporal reward discounting tasks. Temporal discounting (TD) may be defined as the decrease of the subjective value of a (often monetary) reward as a function of pre-reward delay durations (Rachlin, 1989; Ainslie, 1992; Green and Myerson, 1993; Critchfield and Kollins, 2001). Put simply, the subjective value of a reward typically decreases the longer one has to wait for it. The rate at which this discounting takes place is measured by observing preferences when presented with choices between small immediate rewards and larger delayed rewards. In these choices, the large reward is usually kept constant (for example $100), while the small reward varies (e.g., $10/20/30/40/50/60/70/80/90). The delays preceding the large reward are also varied (e.g., 1/30/90/180/365 days). For each delay, the large reward is paired with each of the small rewards and presented as a choice. For example, one choice may be “would you rather receive $50 today or $100 after 30 days?” By observing for each delay how large the immediate reward needs to be for a participant to be indifferent between the two options, one can then plot a discounting function indicating how rapidly the subjective value of a large reward decreases as a function of waiting time (see Critchfield and Kollins, 2001; Scheres et al., 2013). Generally, strong preferences for small immediate rewards result in steep TD functions (small area under the curve), while strong preferences for large delayed rewards result in shallow TD functions (large area under the curve).

The ability/willingness to forego smaller immediate rewards in favor of larger delayed rewards varies across individuals and is especially weak in those with psychiatric conditions with a childhood or adolescence onset, such as Attention-Deficit/Hyperactivity Disorder and Substance Abuse (Sonuga-Barke et al., 2008; de Wit, 2009; Luman et al., 2010). Additionally, the seminal work by Mischel et al. (2011 for a review), which utilizes the classic delay of gratification paradigm, has shown that the ability/willingness to wait at a young age predicts success in the academic, social, cognitive, and emotional domains during adolescence and adulthood. However, while the ability/willingness to wait has been shown to increase from young adulthood to old age (e.g., Green et al., 1994; Jimura et al., 2011; see also Green et al., 1996), our knowledge about how this develops from childhood into adulthood is still limited. This is because studies are relatively few (Scheres et al., 2006; Olson et al., 2007; Steinberg et al., 2009; Prencipe et al., 2011; Demurie, 2012; de Water et al., 2014), task formats vary across studies (hypothetical, potentially real, and real), and the majority of studies only included a relatively small age range (Scheres et al., 2006; Prencipe et al., 2011; Demurie, 2012; de Water et al., 2014), therefore not giving insight into the development from childhood into adulthood. One important reason to understand this developmental pathway in healthy populations is that it will provide a reference with which to compare individuals with childhood/adolescence-onset clinical conditions, such as ADHD and substance abuse.

Thus far, studies examining age effects on TD have primarily relied on tasks in which waiting times do not need to be endured and rewards are not actually paid to participants. Administering such a *hypothetical task* with large rewards of $1000 and delays ranging from 1 day to 1 year to 935 adolescents and adults, Steinberg et al. (2009) reported that individuals aged 13 and younger discounted delayed rewards significantly more steeply than individuals aged 16 and older, with the 14–15 years olds falling in between. Similarly, Christakou et al. (2011) reported a linear increase from adolescence to adulthood in ability/willingness to wait for the large reward in a hypothetical TD paradigm with $100 as the large reward and delays between 1 week and 1 year. Finally, Demurie (2012) used a hypothetical TD task with smaller money amounts and shorter waiting times: the large reward was 30 euro, and waiting times varied between 1 day and 2 weeks. They observed an increase in ability/willingness to wait for 30 euro from 8–10 to 11–13 years old, but the 14–16 years olds fell somewhere in between the 8–10 and 11–13 years olds. This suggests an inverted U-shaped relation between age and ability/willingness to wait, with maximal ability/willingness to wait in 11–13 years olds.

Four studies examining age effects on TD made use of *potentially real tasks*. In these tasks, participants are informed that one choice will be randomly selected and paid to the participant (Olson et al., 2007; Prencipe et al., 2011; de Water et al., 2014), or that there is a one in six chance that one choice will be randomly selected and paid to the participants (Audrain-McGovern et al., 2009). This design relies on the assumption that participants will choose on each trial pair as if it will have real consequences. In three of these studies, the large reward was $10 and delays preceding this reward ranged from 1 day to 1 year. A linear increase in ability/willingness to wait from 8–9 years of age to 14–15 years of age was observed (Prencipe et al., 2011), and from adolescence to adulthood (Olson et al., 2007; de Water et al., 2014). In the third study that used a potentially real task (Audrain-McGovern et al., 2009), the large reward varied between $25 and 85. The delays preceding this reward varied between 1 week and 6 months. Additionally, this study was unique in that it used a longitudinal (as opposed to cross-sectional) design, following participants from age 15 to 20, with data collected once every year. TD was found to be stable.

Finally, only in one study by Scheres et al. (2006), a *real, experiential task* was used, with large rewards of 10 dollar cents and actual waiting times between 5 and 30 s. Delays were endured by participants and money amounts were really paid to participants. With this task, Scheres et al. reported that 6–11 years olds discounted delayed rewards more strongly than 12–17 years old, suggesting a linear increase in the ability/willingness to wait from childhood into adolescence.

Taking these studies together, we can conclude that there is a clear need for studies with more ecologically valid tasks in which a wider age range is included in order to enable researchers to examine whether decreases in discounting from childhood to young adolescence persist into older adolescence and emerging adulthood, or whether non-linear patterns may be observed from childhood into young adulthood. When summarizing the results from current studies, we can conclude that findings are mixed: age effects were reported to be linear, non-linear, and non-existent. Explanations for these inconsistencies may be found in the fact that most researchers may only have examined linear effects and not non-linear effects as well, in the different age ranges used, and in potentially meaningful differences in the specific TD task parameters such as magnitude of the rewards, duration of the delays, and whether the task was hypothetical, potentially real, or real. Most studies relied on hypothetical or potentially real tasks, limiting the ecological validity. The age range in most studies was restricted: only children and adolescents were included, or adolescents and adults, while only two studies enrolled children, adolescents, as well as adults (Olson et al., 2007; Steinberg et al., 2009). These latter two studies were limited in terms of ecological validity because they did not use real tasks. Previous work has shown that changes in task parameters and format have significant effects on the degree of discounting (Robles and Vargas, 2007, 2008; Tesch and Sanfey, 2008; Robles et al., 2009; Rodzon et al., 2011). Therefore, it is likely that differences in results are partly related to task differences.

More specifically, one may hypothesize that the findings depend on the relative contribution of self-control vs. affective processes to the choices made during the specific task. Although traditionally, the ability/willingness to wait for a large reward has been viewed as a sign of self-control, and a relative preference for small immediate rewards is often considered an index of impulsivity (e.g., Critchfield and Kollins, 2001; Green and Myerson, 2004), we suggest that TD may be better viewed as reflecting a trade-off between the ability to wait (self-control), and affective processes such as the sensitivity to the immediacy of the small reward and sensitivity to the magnitude of the delayed reward. Concretely, in order to wait for the larger delayed reward, an individual must be both willing and able to endure the waiting time, and be sufficiently motivated by/interested in the large reward as compared to the smaller, immediate alternative, to make the waiting worthwhile (see Marco et al., 2009; Kable, 2010; Scheres et al., 2010a for related discussions). Importantly, the relative contribution of self-control functions and affective processes in TD tasks may vary depending on the specific task parameters chosen. For example, in hypothetical tasks and/or in tasks with long delay durations, affective processes may play a minor role while cognitive control function may play a primary role. Therefore, the linear age effects which were mostly reported in studies with hypothetical tasks and long delay durations might be due to improvements in cognitive control functions during adolescence (Ridderinkhof and van der Molen, 1997; Williams et al., 1999; Luna et al., 2004; van den Wildenberg and van der Molen, 2004; Luciana et al., 2005; Huizinga et al., 2006; Bunge and Wright, 2007; Crone, 2009; Mischel et al., 2011). Whether studies with real tasks and/or short delay durations, which may rely more heavily on affective processes, will find linear or non-linear age effects in a sample spanning childhood to adulthood is an empirical question.

Therefore, the current study had three primary aims. First, we focused on a relatively wide age range, examining age effects on TD by including children, adolescents, as well as emerging adults. To this end, we included data from participants in these three age groups that were previously collected as part of larger studies (Scheres et al., 2006, 2008, 2010a,b). All participants were administered the same task. Secondly, we chose to use a *real task* in which participants experienced the consequences of their choices during the experiment: They endured the waiting times and received the rewards (money amounts displayed on the screen, and the actual sum of money paid at the end of the experiment). This is a clear advantage in terms of ecological validity, especially for the age range used here (see Navarick, 2004; Reynolds and Schiffbauer, 2004; Lagorio and Madden, 2005; Scheres et al., 2010b). While in real tasks, the reward magnitudes are necessarily small (typically up to $1) and delay durations relatively short (up to 60 s), these tasks have been proven to be useful in examining individual differences in children and adolescents. Additionally, the task is also a sensitive measure of discounting in young adults, as ceiling/floor effects have not been shown (Scheres et al., 2010b). The third aim of this study was to examine both linear and non-linear age effects on discounting.

Due to inconsistent findings in prior research, and based on different theoretical frameworks, several opposing hypotheses could be formulated. Discounting might be hypothesized to decrease linearly with age from childhood to adolescence and emerging adulthood, based on previous findings (Scheres et al., 2006; Olson et al., 2007; Steinberg et al., 2009), and based on the assumption that TD tasks primarily tap into cognitive control abilities which follow a protracted developmental pattern (Ridderinkhof and van der Molen, 1997; Williams et al., 1999; Luna et al., 2004; van den Wildenberg and van der Molen, 2004; Luciana et al., 2005; Huizinga et al., 2006; Bunge and Wright, 2007; Crone, 2009; Mischel et al., 2011). In contrast, non-linear age-related changes in TD, with a peak ability/willingness to wait during adolescence could be predicted based on one previous study (Demurie, 2012), and based on the notion that tasks with short delays and real rewards may trigger a relatively heavy involvement of affective processes, which play a unique role during adolescence (Ernst et al., 2005, 2006; Steinberg, 2005, 2008, 2010; Galvan et al., 2006; Casey et al., 2008a,b; Somerville et al., 2010; van Leijenhorst et al., 2010a,b; Crone and Dahl, 2012). A third possibility is that discounting remains stable, although previous work reporting on the lack of age effects was limited to 15–20 years olds (Audrain-McGovern et al., 2009). In order to address these competing hypotheses, we tested both linear and non-linear (i.e., quadratic) age effects in this study.

## Materials and methods

### Participants

Three groups participated: children, adolescents, and young adults. The child group consisted of 39 typically developing 6–12 year olds (25 male, 14 female; mean age 9.3, *SD* = 1.9), and the adolescent group consisted of 28 typically developing 13–17 year olds (17 male, 11 female; mean age 14.4, *SD* = 1.2). Both of these groups participated in previous research in which they were compared to children and adolescents with ADHD (Scheres et al., 2006, 2010a). The young adult group consisted of 55 undergraduate psychology students (26 male, 29 female; mean age 18.2, *SD* = 0.4) (see Scheres et al., 2008, 2010b).

### Task

**Figure 1:**
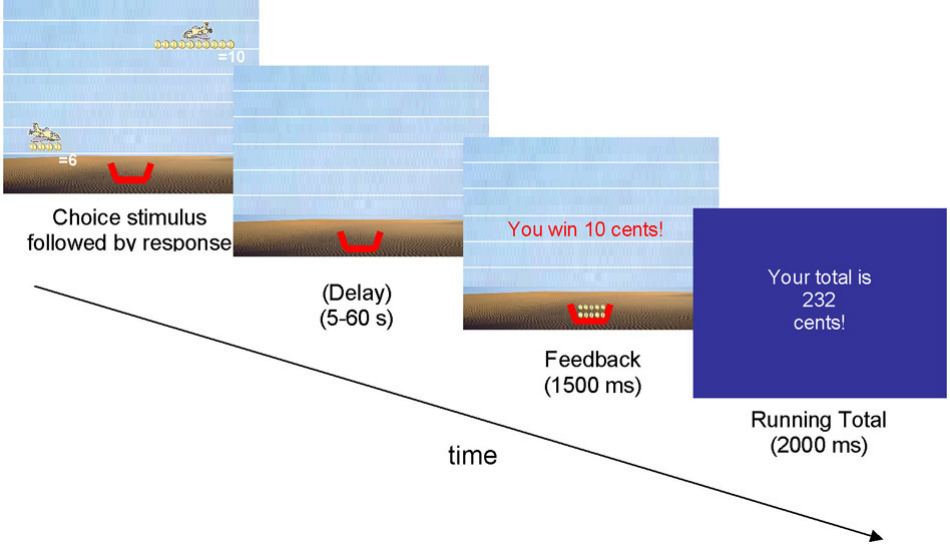
**Example of a temporal reward discounting choice trial**. This choice is between 6 cents immediately and 10 cents after 30 s.

Participants played a TD task in which they were instructed to make repeated choices between a small variable reward (2, 4, 6, or 8 cents) that would be delivered after 0 s and a large constant reward (10 cents) that would be delivered after a variable delay [5, 10, 20, 30, (or 60) s]. For example, on some trials, participants had to choose between 6 cents now or 10 cents after waiting 20 s. Trials were administered in the same pseudo-random order to all participants. Each small immediate reward was paired twice with every delay for the large reward, resulting in a total of 40 choice trials. Choices were visually represented by two airplanes on a computer screen; each airplane carried their corresponding quantity of money, which was represented by a number and that specific amount of coins. Delays were represented by the level that the airplanes were flying—the higher the plane, the longer the delay duration (see Figure 1). The left or right position of the delayed reward plane was balanced over trials. Participants made a choice by pressing the button corresponding to the preferred plane (right or left) which resulted in the plane “dropping” its money into the participant's money basket on the bottom of the computer screen, either immediately or after the appropriate delay. Before the next trial the computer visually updated the total number of cents won. Participants were informed of the number of trials they would be presented with. Importantly, participants were not informed about the durations of the delays. Instead, they endured each delay during task practice, giving them a sense of the delay duration associated with each airplane level, without revealing the actual duration. After task completion, participants received the total amount of money won.

### Procedure

The study was approved by the Human Subjects Protection Program of the University of Arizona and New York University School of Medicine, and all participants provided prior written informed assent and consent.

### Data preprocessing

Data were preprocessed based on previously reported procedures (Myerson et al., 2001; Scheres et al., 2006). Two independent raters determined subjective values for the delayed reward for each delay. Subjective value was defined as the magnitude of the small immediate reward for which the participant showed indifference in a choice against the large delayed reward (see Critchfield and Kollins, 2001; Scheres et al., 2006). Between-rater agreement was very good (mean kappa 0.90, range 0.72–1.00). In rare cases of disagreement, a consensus on subjective value was reached by discussion. The second step was to calculate area under the discounting curve (AUC) for the TD functions (following the procedure described by Myerson et al., 2001, and used by Scheres et al., 2006, 2010a,b). First, subjective values and delays were normalized. That is, subjective values were expressed as proportions of the amount of the maximum delayed reward, and delays were expressed as proportions of the maximum delay. In this case, since the delays ranged from 5–30 in one dataset (Scheres et al., 2006) and from 5–60 in the other datasets (Scheres et al., 2010a,b), 30 s was used as the maximum delay for the purpose of delay standardization (see Myerson et al., 2001). The normalized values were used as *x* and *y* coordinates (*x* = delay; *y* = subjective value). The data points on the y axis were connected, thus forming the discounting function. From each standardized subjective value, vertical lines were drawn to determine separate trapezoids. The area of each trapezoid equals (*x*2 − *x*1) * [(*y*1 + *y*2)/2], where *x*1 and *x*2 are successive delays, and *y*1 and *y*2 are the subjective values associated with these delays. Using this formula, the area of each trapezoid was calculated and subsequently the areas were summed, resulting in the dependent variable of interest: total AUC which has a range of 0–1. In general, a smaller AUC reflects a steeper discounting function (i.e., less willingness to wait as the delay duration increases).

### Statistical analyses

#### Age group comparisons

ANOVAs were conducted with AUC as the dependent variable and age group with three levels (children, adolescents, emerging adults) as the between subject factor. Significant main effects of group in the ANOVA were followed by contrast analyses to examine the shape of the age effect.

#### Regression analyses

Because the group stratification by age may be viewed as arbitrary, we also used linear regression to examine the continuous relation between age and AUC. In order to examine linear effects, age was entered as the predictor and AUC as the dependent variable. In order to test for non-linear (quadratic) age effects, age × age (after centering age around its mean) was entered as a predictor, with AUC as the dependent variable. Finally, in order to test whether there was a meaningful difference in the explanatory power of linear vs. quadratic effects, age was entered as a predictor at step 1, followed by age × age at step 2.

## Results

**Figure 2:**
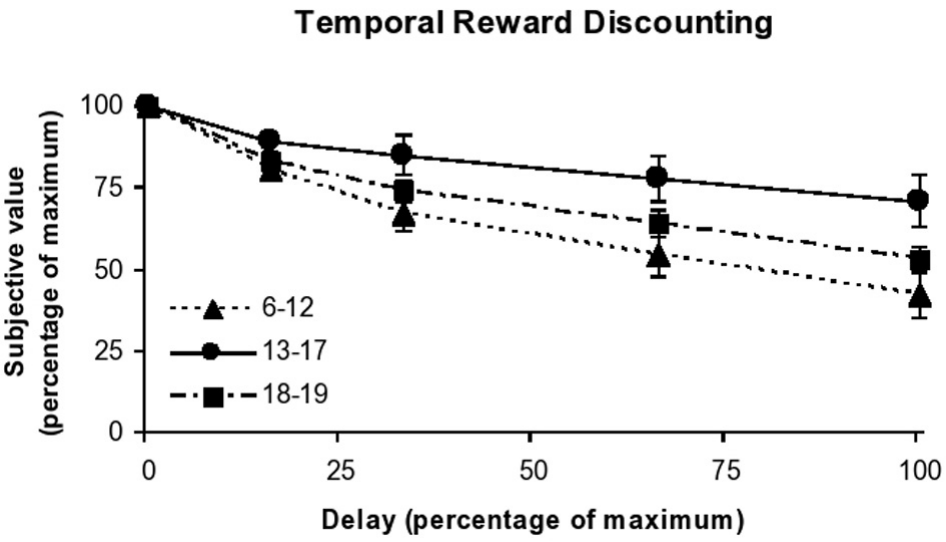
**Temporal reward discounting functions for children, adolescents, and young adults**.

ANOVA showed that there was a significant, medium-sized effect of age group on AUC when comparing children, adolescents, and adults [*F*_(2, 121)_ = 7.4, *p* < 0.001; partial η^2^ = 0.11]. Subsequent contrast analysis revealed that the shape of this age effect was best described as quadratic (*p* < 0.001, as compared to *p* < 0.08 for linear), with an increase in AUC from childhood (*M* = 0.49, *SE* = 0.03) to adolescence (*M* = 0.67, *SE* = 0.04), followed by a decrease in AUC from adolescence to emerging adulthood (*M* = 0.56, *SE* = 0.03). Figure 2 displays the discounting curves for each age group.

**Figure 3:**
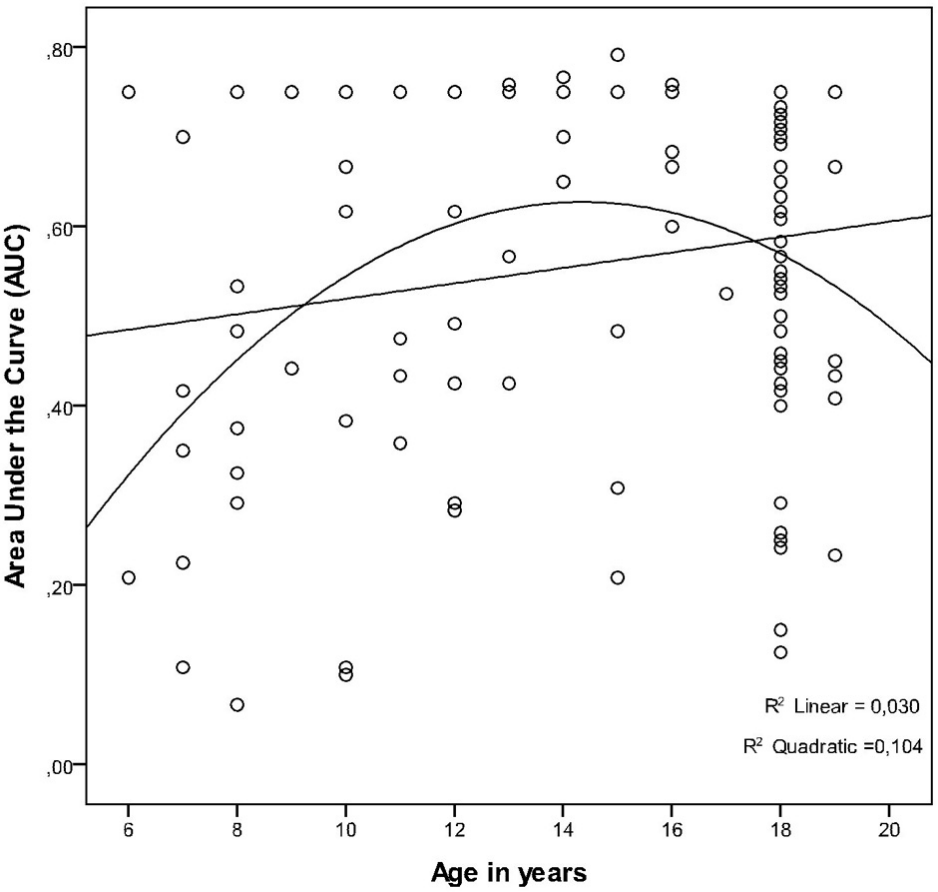
**The relation between age and ability/willingness to wait, as measured by Area Under the discounting Curve (AUC)**.

Regression analysis showed that there was a small-sized linear increase of AUC with increasing age that fell short of statistical significance (*r* = 0.17; *p* < 0.058, *R*^2^ = 0.03). The quadratic relation between age and AUC, however, was significant and medium-sized (*r* = −0.32; *p* < 0.001, *R*^2^ = 0.10). The ability/willingness to wait appeared to peak around age 14 (see Figure 3). The quadratic effect added explanatory power over and beyond the linear effect (Δ *R*^2^ = 0.08; *p* < 0.002).

## Discussion

This study had as a goal to examine whether age effects on temporal reward discounting (TD) with a *real task* with relatively small money amounts and short waiting times were linear or quadratic in a group of participants spanning childhood to emerging adulthood (6–19). To this end, we analyzed data from a real TD task that was administered to children, adolescents, and young adults (Scheres et al., 2006, 2008, 2010a,b). We tested both linear and quadratic effects of age on AUC. The findings did not support the hypothesis of a linear relation between age and TD, but did support the hypothesis that of a quadratic relation between age and TD, with the ability/willingness to wait for large rewards peaking during adolescence, in particular at the age of 14.

At first, this may seem a counter-intuitive finding because adolescents are often thought of as impulsive, while choosing to maximize rewards relatively often as they did on this task is typically viewed as the opposite of impulsive, namely as a sign of self-control (e.g., Critchfield and Kollins, 2001; Green and Myerson, 2004; Mischel et al., 2011). For instance, Mischel et al. (2011) have proposed that the ability to resist temptation in favor of long-term goals, also referred to as “willpower,” self-control, or a variety of inhibitory processes, is the key ingredient which determines to what extent a participant is able to overcome a tempting immediate reward (see also Figner et al., 2010). However, we note here that inhibitory control has been shown to develop linearly from childhood into adulthood (Ridderinkhof and van der Molen, 1997; Williams et al., 1999; Crone, 2009). On the contrary, our data show a non-linear developmental pattern for TD. Thus, we suggest here that multiple processes are important determinants of choice preference in TD tasks, with inhibition being only one of them. The following factors also play an important role: sensitivity to reward immediacy, sensitivity to reward magnitude, and delay aversion (see Marco et al., 2009; Scheres et al., 2010a).

Therefore, based on the idea that it is useful to think of self-control and various reward aspects as playing complementary and interactive roles in TD paradigms, we propose the following interpretation of the data: Choices made on TD paradigms likely result from a balance between affective processes including delay aversion and sensitivity to various reward aspects (such as immediacy of the reward, and magnitude of the reward) on the one hand, and self-control on the other. This balance may be biased toward affective, reward-related aspects or toward self-control, depending on factors such as age and context (e.g., is the task real or hypothetical). Our data show that the balance gravitated toward “self-control” in adolescence, as compared to childhood and adulthood. However, this does not necessarily mean that self-control peaks in adolescence: On the contrary, there is ample evidence to suggest that self-control keeps improving well into adulthood (Williams et al., 1999; Crone, 2009). Rather, the current findings may suggest that a relatively high sensitivity to reward magnitude/maximization motivated adolescents to wait, despite their generally immature ability to exert self-control. This interpretation is consistent with previous research which has demonstrated that reward-seeking or reward sensitivity peaks during early to middle adolescence (Galvan et al., 2006; Steinberg, 2008, 2010; Casey et al., 2008a,b), and that reward- and emotion- related brain regions (for example, the nucleus accumbens) are relatively mature during adolescence (e.g., Casey et al., 2008a,b; Steinberg, 2008; see also Ernst et al., 2005, 2006; Galvan et al., 2006; Galvan, 2010; Steinberg, 2010; van Leijenhorst et al., 2010a,b). More generally, it is consistent with the idea that early to middle adolescence is a unique developmental period.

Alternatively, it may be suggested that rather than the ability to exert self-control being statically immature during adolescence, this ability may flexibly fluctuate, depending on contextual, social, and emotional factors. In this case, it may be hypothesized that adolescents exerted great self-control because they were highly motivated to maximize their financial gains (see Crone and Dahl, 2012 for a recent theoretical framework). In a different context, such as in a hypothetical task in which the money would not be paid to the participants, the motivation to exert self-control may be lower, and a different developmental pattern for TD may be found. This may be a plausible explanation for the fact that most studies with hypothetical or potentially real TD tasks have reported linear effects of age on the ability/willingness to wait (Olson et al., 2007; Steinberg et al., 2009; Christakou et al., 2011; Prencipe et al., 2011; de Water et al., 2014).

More generally, we cannot assume that hypothetical and real TD tasks measure similar processes. Specifically, Navarick (2004) has suggested that hypothetical TD tasks may measure an entirely different discounting process than tasks using real rewards and real delays. However, note that experimental research has later demonstrated that previously found differences in discount rate between real and hypothetical tasks are attributable to differences in reward magnitudes and delay durations (Lagorio and Madden, 2005; Scheres et al., 2010b). It will be of interest to directly compare the developmental pattern of real and hypothetical discounting tasks with varying reward magnitudes and delay durations in future research. Additionally, adding tasks that measure self-control functions such as response inhibition and working memory, as well as tasks that tap into affective aspects such as reward valuation, will provide further insights.

A number of limitations need to be considered here. The possible interpretations of the quadratic age effect are still speculative, and future research is clearly needed to replicate and clarify these findings, including functional brain imaging work to determine whether these age effects can be in part explained by neuromaturational effects. Secondly, this study was aimed at exploring the developmental pattern of TD from childhood into young adulthood but did not include additional measures. We recommend that future research includes a behavioral measure of money valuation, to test whether the decrease in discount rate from adolescence to adulthood can be explained by differences in money valuation. Additionally, future research may implement task manipulations to specifically examine the relative contribution of the various reward aspects in this task such as sensitivity to reward immediacy, reward magnitude, and delay aversion (see Scheres et al., 2010b). More specifically, such research could include multiple versions of a TD task, such as with and without post-reward delays (see Sonuga-Barke et al., 1992; Scheres et al., 2006; Marco et al., 2009), and with varying reward magnitudes and number of trials (Scheres et al., 2010a) in order to achieve this goal. Finally, our goal is to follow this study by a larger lifespan development study on TD, which will provide us with a reference with which to compare the TD development of clinical populations, such as ADHD (see Paus et al., 2008).

In conclusion, this study showed that the cross-sectional development of TD from childhood into young adulthood as measured with a *real task*, follows a quadratic pattern. More specifically, the ability/willingness to wait peaked at age 14. Various interpretations of this finding were offered, including a focus on reward maximization despite an immature ability to exert self-control, and a high level of self-control which was motivated by the prospect of a maximum money amount. These data may provide a reference with which to compare the development of TD in clinical populations. Additional task manipulations as well as the use of functional brain imaging will help to further clarify the nature and biological basis of this finding.

### Conflict of interest statement

The authors declare that the research was conducted in the absence of any commercial or financial relationships that could be construed as a potential conflict of interest.
